# Persistent Positive Real-Time Reverse Transcription Polymerase Chain Reaction (RT-PCR) Results in Recovered COVID-19 Hospital Patients: Implications for Interpretation

**DOI:** 10.7759/cureus.50029

**Published:** 2023-12-06

**Authors:** Jian Hur, Dong-Min Kim, Donghwi Park, Min Cheol Chang

**Affiliations:** 1 Department of Infectious Diseases, Yeungnam University Medical Centre, Daegu, KOR; 2 School of Medicine, Chosun University, Gwangju, KOR; 3 Department of Physical Medicine and Rehabilitation, Ulsan University Hospital, Ulsan, KOR; 4 Department of Physical Medicine and Rehabilitation, Yeungnam University Medical Centre, Daegu, KOR

**Keywords:** respiratory symptom, polymerase chain reaction, culture, covid-19, cough, coronavirus

## Abstract

Background

Real-time reverse transcription polymerase chain reaction (RT-PCR) test results often remain positive in patients with COVID-19, even after their symptoms have improved. We compared the characteristics of patients with persistently positive RT-PCR test results despite improved COVID-19 symptoms to those whose RT-PCR test results turned negative following symptom improvement.

Materials and methods

A total of 143 adult patients with COVID-19 who were hospitalized at a tertiary university hospital were enrolled. Demographic, clinical, treatment, and epidemiological data were extracted from their electronic medical records. These data were compared between patients with persistently positive RT-PCR test results and those with negative RT-PCR test results after symptom improvement.

Results

The prevalence of cough and respiratory symptoms was less in COVID-19 patients with persistently positive RT-PCR test results after symptom improvement than in other patients with COVID-19 (p<0.05).

Conclusion

Persistently positive patients had a lower prevalence of cough than those who became negative. None of the other examined co-variates (hypertension, chronic kidney disease, chronic lung disease, dyslipidemia, etc.) was associated with the persistent positivity.

## Introduction

On March 11, 2020, the WHO declared the outbreak of COVID-19 to be a global pandemic. Since then, COVID-19 has spread to over 200 countries [1]. To identify patients with COVID-19, a real-time reverse transcription polymerase chain reaction (RT-PCR) test, which extracts, amplifies, and detects specific genes of the new SARS-CoV-2, is being used [2]. The RT-PCR test detects the presence or absence of a specific gene in a sample taken from patients' nasal cavities and pharynx [2]. The advantage of this test is that it takes as little as six hours to obtain the results [2]. However, false-negative results may appear due to various problems such as virus mutation, PCR reagents or equipment efficiency, and extraction quality. Furthermore, while the cycle threshold (Ct) value of the amplification of a template can be detected, there is no absolute Ct cut-off value to differentiate between positive and negative results [2]. Therefore, the classification of positive and negative results remains unclear, and false-negative and false-positive results are often yielded [2, 3]. Moreover, given that mutations occur frequently in SARS-CoV-2, there has been the possibility of false negatives in the diagnosis of COVID-19 because RT-PCR methods usually detect only 2-3 of these genes. In previous studies, researchers found that the median duration of SARS-CoV-2 presence in stool samples (22 days) was significantly longer than in respiratory (18 days) and blood samples (16 days). They also discovered that the median duration of the virus in respiratory samples from patients with severe disease (21 days) was significantly longer than in those with mild disease (14 days) [4]. In the mild group, viral loads peaked in respiratory samples during the second week from disease onset, while in the severe group, viral load remained high during the third week [4]. Therefore, the PCR test may return a negative result depending on the sample's location, even though the virus is actually present in the body. Another disadvantage of the RT-PCR test is that residual virus gene fragments can yield a false-positive result even after the virus dies [5]. Even if COVID-19 patients have recovered and have no potential to infect other people, the RT-PCR test may still yield a positive result. However, these results can be supplemented by additional tests such as virus culture. RT-PCR test results are often positive, even in patients whose COVID-19 symptoms have improved. Therefore, it can be difficult to decide whether to discharge a patient from quarantine due to the possibility that live SARS-CoV-2 remains in the body is still present. The underlying cause of this should be clarified so that appropriate guidelines for managing patients with positive RT-PCR results even after symptom recovery can be provided. Further research on the characteristics of patients with consistently positive RT-PCR test results despite symptom improvement may help clarify the cause and prepare countermeasures. To aid in the clinical interpretation of persistent positive RT-PCR test for COVID-19, we compared the characteristics of patients with persistently positive RT-PCR test results for COVID-19 to those with negative RT-PCR test results despite symptom improvement. In addition, a virus culture test was performed in patients with persistently positive results.

## Materials and methods

Study design and participants

This retrospective cohort study included adult inpatients (≥19 years old) from Yeungnam University Hospital in Daegu, South Korea. We screened all adult patients diagnosed with COVID-19 according to WHO interim guidance. The study encompassed those admitted to Yeungnam University Hospital from February 11, 2020 (when the first patients were admitted) to June 15, 2020. However, since there were not many patients with repetitive positive RT-PCR, sample size calculation could not be performed. Consequently, we included all retrospective data of COVID-19 patients admitted to Yeungnam Hospital. This study was approved by the Institutional Review Board of Yeungnam University Hospital, and the requirement for informed consent was waived by the Ethics Commission, as described previously.

Data collection

Demographic, clinical, treatment, and epidemiological data were extracted from the electronic medical records. All data were investigated by two physicians using a standardized data collection form. Any discrepancies were resolved by discussion.

Laboratory procedures

All patients underwent blood testing within 48 hours of admission. For results to be accurate, after fasting for at least 8 hours, blood samples (WBC, neutrophil percentage, platelet count, C-reactive protein, plasma creatinine, and lactate dehydrogenase) were drawn from the antecubital vein into vacuum tubes and subsequently analyzed at certified laboratories in Yeungnam University Hospital. Drawing blood in a fasting state helps to maintain stable blood glucose.

Virus culture

Virus isolation was attempted using sputum and nasopharyngeal swab samples in viral transport medium from eight COVID-19 patients who exhibited prolonged positive RT-PCR results. The samples were treated with 20x penicillin/streptomycin (Fisher Scientific, Loughborough, United Kingdom; Cat# 15140-122) at 4℃ for one hour, followed by centrifugation at 3,000 rpm for 20 minutes [6, 7]. The supernatant containing the virus was collected and used to infect Vero E6 cells. Subsequently, viral proliferation was confirmed based on a Ct value of 20 using real-time RT-PCR after two passages at 5-day intervals [6, 7].

Virus RT-PCR

After January 31, 2020, the local government's Public Health and Environmental Research Institutes began diagnosing COVID-19 using the RT-PCR kit approved by the Korea Center for Disease Control and Prevention (KCDCP) and the Korean Ministry of Food and Drug Safety [2]. The RT-PCR test for COVID-19 requires two separate assays over a 24-hour period, using nasopharyngeal and oropharyngeal swabs. A positive SARS-CoV-2 detection from respiratory specimens using RT-PCR confirmed the diagnosis of COVID-19. The tests were performed using the BioCore 2019-nCoV Real-Time PCR Kit (Kogene Biotech, Inc., Seoul, Republic of Korea), which has a sensitivity and specificity of 95% and 97%, respectively. All RT-PCR tests in this study were conducted by the Department of Diagnostic Examination Medicine at Yeungnam University Hospital.

Risk factors

Based on previous research on the risk factors of COVID-19 and the classification system announced by the KCDCP, the presence of chronic underlying diseases, such as chronic kidney disease (CKD), diabetes, carcinoma, chronic lung disease, cardiovascular disease, hypertension, and dyslipidemia were investigated [8]. Chronic lung disease was defined as patients with asthma, chronic obstructive pulmonary disease, idiopathic pulmonary fibrosis, bronchiectasis, or interstitial lung disease [9]. Additionally, we investigated the presence of neurological disorders, such as Alzheimer's disease in patients with COVID-19. We also investigated the presence of symptoms, such as fever, chills, cough, dyspnea, sore throat, sputum, rhinorrhea, myalgia, headache, and diarrhea during the infection state. If patients with COVID-19 presented with rhinorrhea, sore throat, cough, sputum, or dyspnea, they were classified as having nasopharyngeal and respiratory symptoms. If patients had none of these five symptoms, then they were classified as without nasopharyngeal and respiratory symptoms.

Radiological factors

We investigated pulmonary infiltration using chest radiographs and chest computed tomography. Pulmonary infiltration was classified as patchy, confluent, or nodular, and unilateral or bilateral by at least two physicians in Yeungnam University Hospital. Furthermore, we used chest radiographs to investigate whether pulmonary infiltration had improved before they were discharged home. A radiologist checked and confirmed the radiological findings.

Other clinical information

We investigated the treatments received by patients, including oxygen therapy, high-flow nasal cannula, mechanical ventilation, and extracorporeal membrane oxygenation (ECMO), along with medications administered such as lopinavir/ritonavir, hydroxychloroquine sulfate, azithromycin, steroids (dexamethasone or prednisolone), and IV immunoglobulin (IVIG) [8, 10]. Additionally, we assessed the presence of severe pneumonia and the National Early Warning Score (NEWS) at admission [11, 12]. Severe pneumonia was identified in patients exhibiting clinical signs of pneumonia (fever, cough, dyspnea, fast breathing) coupled with at least one of the following: a respiratory rate exceeding 30 breaths/min, severe respiratory distress, or a SpO2 level below 90% in room air [13]. The NEWS, developed to predict the prognosis of COVID-19 patients, consists of seven parameters aimed at enhancing the early detection and response to clinical deterioration [12].

Statistical analysis

To examine the differences in demographic data between patients with COVID-19 who had persistently positive PCR test results and other COVID-19 patients after symptom improvement, independent t-tests and Pearson's Chi-square tests were performed. When the sample size was less than 5 in a cell, Fisher's exact tests were used. All statistical analyses were conducted using SPSS version 22.0 (IBM Corp., Armonk, NY, USA). A p-value of less than 0.05 was considered statistically significant.

## Results

Patient demographics

A total of 143 adult patients with COVID-19 who were hospitalized at Yeungnam University Hospital before June 15, 2020, were enrolled in this study. Eight patients (8/143; 5.6%) had three persistently positive consecutive PCR test results after symptom recovery. The mean patient age was 60.03 ± 17.09 years, ranging from 21 to 95 years. The sex ratio (male:female) was 1:1.19. Hypertension was the most common comorbidity, affecting 33.8% of the patients, followed by diabetes (19.7%) and cardiovascular disease (6.3%). In patients with persistent positive RT-PCR test results for COVID-19, the mean age was 60.50 ± 17.8 years, and the sex ratio was 1:1.67. Hypertension was the most common comorbidity, affecting 50.0% (n=4) of the patients, followed by diabetes (12.5%, n=1) and chronic lung disease (12.5%, n=1), chronic kidney disease (12.5%, n=1), and smoking (12.5%, n=1) (Table 1).

**Table 1:** Clinical characteristics of patients with COVID-19. CT: Convolutional tomography; IVIG: Intravenous immunoglobulin; ECMO: Extracorporeal membrane oxygenation.

Variable	Total	PCR (+)	PCR (-)	P-value
Total, n (%)	143	8 (5.6)	135 (94.4)	
Age, years	60.0 ± 17.1	60.5 ± 17.8	60.0 ± 17.1	0.823
Gender, n				
Male (%)	64 (44.1)	3 (37.5)	61 (45.2)	
Female (%)	79 (54.5)	5 (62.5)	74 (54.8)	0.671
Obesity (BMI≥30)	35	2	33	0.972
FBS (mg/dL)	137.9 ± 63.9	122.9 ± 44.4	138.7.0 ± 64.8	0.650
White blood count (x10^9^/L)	6.8 ± 3.1	7.4 ± 2.8	6.7 ± 3.1	0.380
Neutrophil (%)	68.1 ± 14.0	68.5 ± 16.1	68.1 ± 13.9	0.986
Platelets (x10^9^/L)	233.0 ± 100.4	233.8 ± 68.7	233.0 ± 102.1	0.968
C-reactive protein (mg/L)	6.6 ± 8.6	6.3 ± 8.7	6.6 ± 8.7	0.270
Plasma creatinine (mg/dL)	1.1 ± 2.4	4.3 ± 9.7	0.9 ± 0.6	0.222
Lactate dehydrogenase (IU/L)	673.9 ± 523.6	552.6 ± 237.8	681.6 ± 536.3	0.400
Comorbidities				
Presence of any comorbidities (%)	48 (33.6)	2 (25.0)	46 (34.1)	0.935
Hypertension (%)	48 (33.6)	4 (50.0)	44 (32.6)	0.319
Diabetes mellitus (%)	28 (19.6)	1 (12.5)	27 (20.0)	0.597
Chronic kidney disease (%)	5 (3.5)	1 (12.5)	4 (3.0)	0.156
Dyslipidemia (%)	6 (4.2)	0 (0.0)	6 (4.4)	0.542
Chronic lung disease (%)	5 (3.5)	1 (12.5)	4 (3.0)	0.156
Malignancy (%)	7 (4.9)	0 (0.0)	7 (5.2)	0.509
Cardiovascular disease (%)	9 (6.3)	0 (0.0)	9 (6.7)	0.449
Dementia (%)	6 (4.2)	0 (0.0)	6 (4.4)	0.541
Smoking (%)	5 (3.5)	1 (12.5)	4 (3.0)	0.156
Intra-hospital infection (%)	11 (7.7)	0 (0.0)	11 (8.2)	0.401
Symptom				
Presence of any symptom (%)	135 (94.4)	8	127	0.479
Fever (%)	95 (70.4)	5	90	0.808
Chilling (%)	27 (18.9)	2	25	0.649
Cough (%)	89 (62.2)	2	87	0.025*
Dyspnea (%)	64 (44.8)	1	63	0.059
Sore throat (%)	24 (16.8)	0	24	0.191
Sputum (%)	71 (49.7)	3	68	0.479
Rhinorrhea (%)	16 (11.2)	1	15	0.904
Myalgia (%)	54 (37.8)	2	52	0.443
Headache (%)	51 (35.74)	1	50	0.159
Diarrhea (%)	26 (18.2)	3	23	0.145
Presence of any nasopharyngeal symptom (%)	113 (79.0)	4	109	0.038*
Severe pneumonia at admission (%)	48(33.6)	1	47	0.186
Severe pneumonia during admission (%)	56 (39.2)	3	53	0.895
Initial early warning score	3.7 ± 3.3	3.3 ± 3.7	3.7 ± 3.3	0.610
Admission day after symptoms onset (days)	9.0 ± 6.5	6.9 ± 3.9	9.1 ± 6.7	0.458
Admission day after diagnosis (days)	4.2 ± 5.8	6.0 ± 10.8	4.1 ± 5.5	0.673
Radiologic findings				
Abnormal findings in chest radiograph (%)	119	7	112	0.875
Abnormal findings in chest CT (%)	95	5	90	0.603
Medications				
lopinavir/ritonavir (%)	123	7	116	0.901
Hydroxychloroquine (%)	128	7	121	0.849
Azythromycin (%)	117	6	111	0.980
Steroid (%)	50	2	48	0.543
IVIG (%)	2	0	2	0.729
Types of intensive care				
Oxygen therapy	54	3	51	0.987
High-flow nasal cannula	25	1	24	0.774
Mechanical ventilator	27	1	26	0.718
ECMO	7	0	7	0.525

Virus culture

In patients with persistent positive RT-PCR test results for COVID-19, there was no growth in virus culture.

Treatment methods

Of the 143 patients, seven (4.9%) were treated with ECMO, 27 (approximately 18.9%) were intubated and placed on a mechanical ventilator, and 26 (18.2%) were treated in the ICU. All hospitalized patients received treatment with either hydroxychloroquine sulfate 400 mg (Oxiklorine®) per day alone, or in combination with lopinavir/ritonavir 200 mg/50 mg (Kaletra®) per day.

Risk factors

In COVID-19 patients with persistently positive PCR test results after symptom improvement, the prevalence of a cough and respiratory symptoms was significantly less than in other patients with COVID-19 (p-value <0.05; Table 1). However, the presence of hypertension, CKD, chronic lung disease, or dyslipidemia was not statistically different between COVID-19 patients with persistently positive PCR test results after symptom improvement and other patients with COVID-19(p≥0.05). And other laboratory results, radiologic findings, medications, and types of intensive care was not statistically different between COVID-19 patients with persistently positive PCR test results after symptom improvement and other patients with COVID-19 (p≥0.05).

## Discussion

In our study, we found that approximately 5.6% of patients with COVID-19 had persistently positive PCR test results after symptom recovery. The number of persistent PCR test-positive patients was too low at 8, which may have affected statistical interpretation or reliability. However, we thought that even a small number of patients with persistent positive RT-PCR test for COVID-19 could be helpful in the clinical interpretation of COVID-19 test results if we could identify the tendencies of these patients. In previous studies, it was reported that COVID-19 patients with mild symptoms, older patients, or patients with elevated SARS-CoV-2-specific CD8 T-cell immune responses were more likely to get persistently positive results for COVID-19 test [14, 15]. However, unlike previous studies, our study compared various factors such as comorbidities, symptoms, radiologic findings, medication, and types of intensive care, as well as laboratory tests. In the results of our study, these COVID-19 patients tended to have a relatively low prevalence of a cough or nasopharyngeal or respiratory symptoms compared to other patients with COVID-19. However, sex, age, duration of admission, and the duration between discharge and the interview were not associated with the prevalence of persistently positive PCR test results after symptom improvement. It is thought that these results may stem from several factors. Firstly, the sensitivity of PCR tests for SARS-CoV-2 could be a contributing issue. The RT-PCR test is known to have limitations, particularly with residual virus gene fragments in the body after the virus has died, which can influence test outcomes [5]. Therefore, in COVID-19 patients with persistently positive PCR tests after symptom improvement, it is thought that dead virus debris in the nasal cavity and pharyngeal wall may affect the results of the PCR test even though the virus is already dead. Particularly, patients lacking cough or nasopharyngeal/respiratory symptoms may discharge fewer virus fragments through coughing or rhinorrhea. As a result, dead virus fragments could remain in the nasal cavity and throat for an extended period. This hypothesis is supported by the majority of these patients not having a recurrence of their symptoms or transmitting COVID-19 to others after discharge. Second, there is the possibility that COVID-19 patients with persistently positive PCR results after symptom improvement have a small amount of live virus present in the nasopharyngeal area. Considering the tendency that patient symptoms are proportional to the virus concentration, the amount of virus may be small in the nasopharynx in patients without a cough and nasopharyngeal or respiratory symptoms. In addition, the RT-PCR results have been reported to be more sensitive than the culture results [16]. Therefore, RT-PCR-positive and culture-negative results may be caused by a low amount of virus present in patients with COVID-19. Indeed, considering that the virus concentration in droplets is essential for its propagation in droplets or airborne transmission [17,18], COVID-19 patients with persistently positive RT-PCR results after symptom recovery might have a low risk of virus propagation due to the small amount of virus present that would cause RT-PCR positive and culture-negative results. However, considering this hypothesis, samples from these COVID-19 patients should be cultured more robustly. For example, bronchial aspiration lavage (BAL) fluid, rather than a nasopharyngeal swab or sputum, could be used. In addition, after discharge from the hospital, additional self-isolation may be necessary to prevent the spread of COVID-19 in the community. However, given the small number of samples in our study, these statistics may not be of much significance. However, until now, there have been few comparative studies of patients whose RT-PCR continued to be positive even after symptomatic improvement, so our study is considered to be sufficiently meaningful as a preliminary study. Through this study, we would like to emphasize the caution about RT-PCR interpretation rather than simple statistical differences. For example, the RT-PCR test detects the presence or absence of only 2-3 specific genes in a sample taken from patients' nasal cavity and pharynx. Therefore, a false-negative result may present if a virus mutation has occurred. In addition, there is no absolute Ct cut-off value to differentiate between positive and false-positive results. The Ct cut-off values are different according to the type of RT-PCR kit. Therefore, the classification of positive and negative results remains unclear, and false-negative and false-positive results are often yielded.

Also, residual virus gene fragments can affect results even after the virus dies. In a few patients with COVID-19, even if COVID-19 patients have recovered and have no potential to infect other people, the RT-PCR test may still yield a positive result. Therefore, clinicians should always keep in mind the caution of interpretation of RT-PCR results. It may also be helpful to consider the time of infection and symptoms together with the results of the RT-PCR test. There were several limitations to this study. First, in this study, there was a small number of COVID-19 patients with persistently positive PCR test results after symptom recovery. Moreover, the limitation of the study is having only eight cases in one arm, which affects the statistical interpretation or confidence. Further studies with large numbers of patients may be more helpful in finding other important co-variates. Second, another precise examination, except viral culture, was not performed. Instead, these patients were discharged after their symptoms had improved, and the culture was negative twice at a one-week interval regardless of the RT-PCR test result, in accordance with government policy guidelines. However, considering the results of this study, more accurate and robust measures (e.g., culture using BAL fluid, sub-genomic RNA, or minus-strand RNA assay) may be necessary to prevent disease propagation. For example, in an RT-PCR test, RT-PCR targeting nucleocapsid total RNA cannot distinguish neutralized input virus from replicating virus [19]. Therefore, additional sub-genomic RNA or minus-strand RNA assays can be helpful in the interpretation of persistent-positive PCR tests for COVID-19. Thirdly, we did not conduct more comprehensive culture tests on COVID-19 patients who continually tested positive via PCR after symptom recovery. Additionally, performing invasive BAL on asymptomatic patients might be deemed unethical. Lastly, we did not conduct follow-up RT-PCR tests post-discharge, adhering to government and KCDCP guidelines [20]. However, further research is necessary to fully understand the reasons behind persistently positive PCR test results post-symptom improvement.

## Conclusions

In our study, we found that approximately 5.6% of COVID-19 patients exhibited persistently positive PCR test results after recovering from symptoms. These persistently positive patients tended to have a lower prevalence of cough compared to those who tested negative. None of the other examined co-variates, such as hypertension, chronic kidney disease, chronic lung disease, or dyslipidemia, were associated with this persistent positivity. To accurately determine the cause of persistently positive PCR test results post-symptom recovery, further studies involving a larger cohort of COVID-19 patients are necessary.
